# An Introduction to the *Toxins* Special Issue on “Bee and Wasp Venoms: Biological Characteristics and Therapeutic Application”

**DOI:** 10.3390/toxins8110315

**Published:** 2016-10-28

**Authors:** Sok Cheon Pak

**Affiliations:** School of Biomedical Sciences, Charles Sturt University, Bathurst, NSW 2795, Australia; spak@csu.edu.au; Tel.: +61-2-6338-4952; Fax: +61-2-6338-4993

Venoms, especially bee venom, have been used since ancient times as a healing treatment for various disorders. The therapeutic value of honey bee venom to improve the quality of life of patients has been acknowledged for over a hundred years. Modern approaches of venomics have allowed for the discovery of venom constituents that have proven to be of pharmacological significance and have opened the way to optimization of therapeutic strategies through the use of active components such as melittin and apamin. Subsequently, the application scope of honey bee venom has been expanding from conventional antinociceptive effect to degenerative diseases of the nervous system. This seems to be due to the properties of venom enzymes and peptides for their natural stability as injectable solutes, their effectiveness in reaching targeted tissues, and their ability to synergize their actions by enhancing cell–cell interactions. Expansion of the therapeutic application of bee and wasp venoms has advanced particularly far in recent years, so this is an opportune time to present this Special Issue on bee and wasp venoms, their biological characteristics and therapeutic application.

The venoms of bees and wasps are complex mixtures of biologically active proteins and peptides, such as phospholipases, hyaluronidase, phosphatase, α-glucosidase, serotonin, histamine, dopamine, noradrenaline, and adrenaline. However, melittin, apamin, and mast cell degranulating peptide are found only in bees, while mastoparan and bradykinin are exclusive to wasps. The recent review article on bee and wasp venoms for their potential therapeutic and biotechnological applications in biomedicine focuses on three major peptides, namely melittin, apamin, and mastoparan [1]. While mastoparan has been studied for its antimicrobial, anti-viral, and anti-tumor properties, melittin and apamin have a broad spectrum of therapeutic applications. To aid the reader in cross-referencing these applications, I present here a listing of the bee venom components used for different disease types and the latest references (Table 1). Interestingly, learning enhancement in animals was benefited exclusively from apamin, probably due to its highly specific mode of action in the brain.

This Special Issue features eleven research papers, for which brief synopses follow. Jung et al. [24] provide details on the underlying mechanism of bee venom’s effect against neuronal cell death. Mitochondrial dysfunction of cells was induced by rotenone which is an inhibitor of the mitochondrial respiratory chain complex and known to induce apoptosis via activation of the caspase-3 pathway. Pre-treatment with bee venom enhanced cell viability and ameliorated mitochondrial impairment in a rotenone-treated cellular model. Moreover, bee venom treatment inhibited the activation of JNK signaling, cleaved caspase-3 related cell death, and increased ERK phosphorylaton involved in cell survival in motor neuron cells.

Kim et al. [25] investigated whether melittin provides inhibition on cholangitis and biliary fibrosis in mice. Authors used a well-established animal model for cholangitis and biliary fibrosis, treated with a chronic feeding of 3,5-diethoxycarbonyl-1,4-dihydrocollidine (DDC). DDC feeding led to increased serum markers of hepatic injury, ductular reaction, induction of pro-inflammatory cytokines, and biliary fibrosis. However, melittin treatment attenuated hepatic function markers, ductular reaction, the reactive phenotype of cholangiocytes, cholangitis, and biliary fibrosis.

Cai et al. [6] present the latest insight into the effect of bee venom acupuncture on ALS. They used hSOD1^G93A^ transgenic mice as an ALS animal model and investigated the effect of bee venom in the central nervous system (CNS) and muscle. Bee venom acupuncture at ST36 enhanced motor function and decreased motor neuron death in the spinal cord compared with that observed in transgenic mice injected ip with bee venom. Furthermore, bee venom acupuncture eliminated signaling downstream of inflammatory proteins such as TLR4, CD14, and TNF-α in the spinal cord, confirming bee venom acupuncture as a chemical stimulant to engage the endogenous immune modulatory system in the CNS, at least in an animal model of ALS. The same group of researchers contributed another research paper [4] to this Special Issue. Given that immune dysfunction of organs from neuroinflammation is a consistent hallmark in ALS, bee venom acupuncture reduced pro-inflammatory proteins in the liver, spleen, and kidney and increased immune response. It was noted that bee venom treatment through an acupoint was more effective than an ip administration of bee venom.

Kang et al. [22] discuss advanced knowledge of the anti-nociceptive efficacy of bee venom. They designed the study to examine the potential anti-nociceptive effect of repetitive bee venom treatment in the development of below-level neuropathic pain in spinal cord injury rats which was induced by spinal hemisection. Hemisection of the rat spinal cord at thoracic level 13 produced prominent mechanical allodynia and thermal hyperalgesia. Repetitive bee venom acupuncture into the Zusanli acupoint on the same side as the spinal hemisection (ipsilateral side) twice a day from 15 to 20 days post-surgery suppressed pain behavior in the ipsilateral hind paw. A spinal hemisection-induced increase in spinal glia expression in terms of glial fibrillary acidic protein and glial ionized calcium-binding adaptor protein 1 was also hindered by repetitive bee venom acupuncture in the ipsilateral dorsal spinal cord. Moreover, bee venom acupuncture facilitated motor function recovery of affected hindlimb.

Li et al. [26] provide therapeutic options for the management of oxaliplatin-induced neuropathic pain. Oxaliplatin produces cold and mechanical hypersensitivity as side effects when used in the treatment of colorectal carcinoma. Their study is a follow-up of a previous evaluation of bee venom alleviating oxaliplatin-induced cold allodynia in rats via noradrenergic and serotonergic analgesic pathways. In the study, authors investigated whether phospholipase A_2_ attenuates oxaliplatin-induced cold and mechanical allodynia in mice. While the significant allodynia signs were observed from one day after an oxaliplatin injection, daily ip administration of phospholipase A_2_ for five consecutive days markedly attenuated cold and mechanical allodynia through the activation of noradrenergic system.

A detailed underlying mechanism of bee venom in ameliorating the renal fibrosis is presented by An et al. [27]. In an animal model of unilateral ureteral obstruction (UUO) for the development of progressive renal fibrosis, bee venom treatment markedly reduced the increased number of infiltrated inflammatory cells with UUO in the kidney tissues. The expression levels of TNF-α, IL-1β, TGF-β1, and fibronectin were significantly reduced in BV-treated mice compared with UUO mice. In addition, the expression of α-SMA was markedly withdrawn after treatment with bee venom. These data suggest that bee venom attenuates renal fibrosis and reduces inflammatory responses by suppression of multiple growth factor-mediated pro-fibrotic genes.

Lee et al. [28] introduce the application of bee venom in porcine reproductive and respiratory syndrome (PRRS) which is a chronic and immunosuppressive viral disease that is responsible for substantial economic losses in the swine industry. Utilizing the immunomodulatory property of bee venom, the study aimed at evaluating the effects of bee venom on the immune response and viral clearance during the early stage of infection with PRRS virus. Bee venom was administered subcutaneously via nasal, neck, and rectal routes, and the pigs were then inoculated with PRRS virus. In experimentally challenged pigs with virus, the viral genome load in the serum, lung, bronchial lymph nodes and tonsil was decreased as was the severity of interstitial pneumonia with bee venom administration. Furthermore, bee venom increased the levels of Th1 cytokines (IFN-γ and IL-12), along with the upregulation of pro-inflammatory cytokines (TNF-α and IL-1β).

Danneels et al. [29] confirm the anti-inflammatory action of *Nasonia vitripennis* venom and the potential anti-cancer role of wasp venom is displayed by the regulation of NF-κB pathway. The possibility of *Nasonia vitripennis* venom as therapeutic agent in some cancers is thus suggested.

Furthermore, Qian et al. [30] characterized two Kazal-type serine protease inhibitors (KSPIs) molecularly in *Nasonia vitripennis*. Most *NvKSPI-1* and *NvKSPI-2* mRNAs were expressed in the venom apparatus. The *NvKSPI-1* and *NvKSPI-2* genes were cloned into vector, and the recombinant products fused with glutathione S-transferase (GST) were purified. When tested against serine protease inhibitors, only GST-*NvKSPI-1* inhibited the activity of trypsin. In addition, both GST-*NvKSPI-1* and GST-*NvKSPI-2* inhibited prophenoloxidase activation in host hemolymph.

Matysiak et al. [31] confirm the influence of bee stings on the serum peptidome profile that broadens the understanding of the human organism’s response to venom thanks to the mass spectrometry-based technique.

Additionally in this issue, there are five review articles. Lee et al. [32] provide current understanding of the mechanisms of the anti-inflammatory properties of bee venom and its components in the treatment of liver fibrosis, atherosclerosis, and skin disease.

Silva et al. [33] present the latest understanding of the mechanisms of action and future prospects regarding the use of new drugs derived from wasp and bee venom in the treatment of some neurodegenerative disorders such as Alzheimer’s disease, Parkinson’s disease, epilepsy, multiple sclerosis, and ALS. Given the severe adverse reactions of crude wasp venom, denatured wasp venom shows an antiepileptic effect by interacting with GABA receptors. Bradykinin isolated from wasp venom protects against ischemic brain injury and promotes neuronal survival.

Hwang et al. [34] discuss the scientific evidence of the therapeutic effects of bee venom and its components on allergic, autoimmune, inflammatory, and neurological diseases. Due to both therapeutic and allergy causing effects of bee venom, the optimal dose and treatment method without adverse reactions should be determined in each disease.

Moreau and Asgari [35] present the latest research on constituents from parasitoid wasp venom with an emphasis on their biological function, applications, and new approaches used in venom studies.

Finally, Moreno and Giralt [1] report on the practical applications of three valuable peptides from bee and wasp venoms. The antimicrobial properties of melittin from bee venom and mastoparan from wasp venom have been the most studied and developed among the components of venoms. Their mode of action on membrane has been developed into antimicrobial peptides, and they can be accepted as a unique solution to the growing incidence of antibiotic resistance.

It is my hope that this Special Issue will be a source for many years to those interested in the agricultural and pharmacological relevance of bee and wasp venoms. The currently available state-of-the-art approaches in proteomics and transcriptomics provide us with intricate mechanisms to further the application of venoms into diverse biomedicine.

## Figures and Tables

**Table 1:** Application of honey bee venom components on different disease types.

Disease Type	Component	Reference
Parkinson’s disease	Bee venom (BV) Apamin BV acupuncture	[2] [2] [3]
Amyotrophic lateral sclerosis (ALS)	BV Melittin BV acupuncture	[4] [5] [4,6]
Multiple sclerosis	BV	[7]
Cancer	BV Melittin	[8] [9]
Liver fibrosis	BV Apamin Melittin PLA_2_	[10] [11] [12] [13]
Atherosclerosis	BV Apamin Melittin	[14] [15] [16]
Skin disease (acne vulgaris)	BV Melittin	[17] [18]
Skin disease (atopic dermatitis)	BV	[19]
Learning deficit	Apamin	[20]
Pain	BV BV acupuncture	[21] [22]
Lupus nephritis	BV	[23]
